# Telemedicine experience of NYC Internal Medicine residents during COVID-19 pandemic

**DOI:** 10.1371/journal.pone.0246762

**Published:** 2021-02-08

**Authors:** Chia-Yu Chiu, Amara Sarwal, Muzamil Jawed, Venkata Sireesha Chemarthi, Nehad Shabarek

**Affiliations:** Department of Internal Medicine, Lincoln Medical Center, New York City, New York, United States of America; Harper University Hospital, UNITED STATES

## Abstract

**Background:**

The COVID-19 pandemic challenged the resilience of public health, including diagnostic testing, antiviral development and transmission prevention. In addition, it also affected the medical education of many residents and learners throughout the country. Historically, physicians undergoing their residency training were not involved in telemedicine. However, in response to the challenges faced due to COVID-19, the Accreditation Council for Graduate Medical Education (ACGME) released a provision in May 2020 to allow residents to participate in telemedicine.

**Method:**

Lincoln Medical Center, located in the South Bronx of New York City, currently has 115 Internal Medicine residents, and telemedicine clinic visits have been conducted by residents since June 2020. An anonymous 25-question survey was sent to all Internal Medicine residents between August 8, 2020 to August 14, 2020.

**Result:**

Of 115 residents, 95 (82.6% of the residents) replied to this questionnaire. Residents revealed feeling less confident in managing chronic diseases through telemedicine visits. The survey also shows that 83.1% of respondents prefer in-person visits during their training, 65.3% feel that the telemedicine experience will affect their future career choice, and 67.4% would prefer less than 50% of visits to be telemedicine in their future careers.

**Outcome:**

The purpose of the new ACGME rules allowing telemedicine was to prevent the undertraining of residents and maintain health care for the patient during the COVID-19 pandemic. This affects residency training and the experiences of residents, which in turn can influence their future career plans.

## Introduction

The term “Telemedicine” was first introduced and described as “delivery of medical care without the usual patient-physician confrontation.” in 1969. Later on, the term “Telehealth” extended the scope of telemedicine by incorporating a “broader set of activities, including patient and provider education in addition to patient care” [1]. However, the dynamic change in technology allowed for the evolution of telemedicine and currently telemedicine not only includes telephone calls, but also video chats and contact via the internet [2]. In 2010, the World Health Organization came up with a broad description of telemedicine, “The delivery of health care services, where distance is a critical factor, by all health care professionals using information and communication technologies for the exchange of valid information for diagnosis, treatment and prevention of disease and injuries, research and evaluation, and for the continuing education of health care providers, all in the interests of advancing the health of individuals and their communities” [3].

On March 18^th^ 2020, the Accreditation Council for Graduate Medical Education (ACGME) issued a letter to the Graduate Medical Education (GME) community entitled “ACGME Response to the Coronavirus (COVID-19)” which immediately permitted residents/fellows to participate in patient care via telemedicine during the COVID-19 pandemic [4]. On March 20^th^ 2020, ACGME issued a “Clarification regarding Telemedicine and ACGME Surveys” emphasizing that “appropriate level of supervision is in place for all residents/fellows based on each resident’s/fellow’s level of education/training and ability, as well as patient complexity and acuity” for these telemedicine visits [5].

In response to the COVID-19 pandemic, ACGME emphasized a three-stage guideline to maintain medical education and patient care: “Stage 1: Business as usual”; “Stage 2: increase clinical demands guidance”; and “Stage 3: pandemic emergency status guidance” [6–8]. In “Stage 1: Business as usual”, direct supervision in telemedicine is defined as “the supervising physician and/or patient is not physically present with the resident and the supervising physician is concurrently monitoring the patient care through appropriate telecommunication technology.” It also allows the resident/fellow to conduct a patient encounter remotely and then discuss the case with the supervising faculty member, also through remote means [6, 9]. “Supervision may include post-hoc review of resident-delivered care with feedback” was also mentioned at ACGME Common Program Requirements (Residency) [9].

Although telemedicine provides the flexibility for patient care during such a time, the feedback from trainees is limited. The purpose of this survey is focused on the trainee response of telemedicine and how confident they were with their medicine practice through this new technique.

## Methods

### Study group

Lincoln Medical Center, located in the South Bronx of New York City, is a full-service medical center and teaching hospital. The Internal Medicine Residency program currently has 115 residents, and telemedicine clinic visits have been conducted by residents since June 2020. Prior to scheduling a patient, medical records are reviewed by attending physicians to decide if patients qualify for telemedicine visits or require an in-person appointment. Within the department of Internal Medicine, telemedicine visits completed by residents occur in Endocrinology, Geriatric, Nephrology, Neurology, Gastroenterology, Cardiology and General Medicine clinics. All telemedicine during that study period is follow-up visit instead of new patient visit. At Lincoln Medical Center, residents conduct telemedicine visits exclusively through telephone visits rather than video calls during the study period. In order to assess the quality and feedback of these visits, an anonymous 25-question survey was sent to all Internal Medicine residents between August 8, 2020 to August 14, 2020.

### Questionnaire design

There are six ACGME Core Competencies which have been used widely to evaluate resident competencies during residency training, including “Provide Patient-centered Care”, “Medical Knowledge”, “Practice-based Learning and Improvement” “Interpersonal and Communication skills”, “Professionalism” and “System-based practice/Work in interdisciplinary teams” [10].

Our questionnaire was designed to target the assessment of these six ACGME core competencies (S1 Data). Questions 1 to 4 are questions related to demographical information, including gender, postgraduate training year and prior COVID-19 experience of telemedicine inside/outside United States. Questions 5, 6, 7, 9, 17 and 21 focus on “Provide Patient-centered Care”. These questions are resident-oriented rather than patient-oriented, which is unique as most studies in literature focus on asking patients directly about their satisfaction. We ask residents how they perceived their patient’s experience to be when encountered virtually. Question 8 is asking how many phone call attempts were made if the patient did not pick up the phone call on the first try. This is important as there are no regulations in regards of how many attempts need to be performed. Questions 10 to 14 focus on “Resident confidence.” We chose some of the common diseases managed in the outpatient setting, including hypertension, congestive heart failure, diabetes, chronic obstructive pulmonary disease/asthma, and chronic kidney disease. We used these questions to assess how confident the trainees are in practicing their medical knowledge through telemedicine. Questions 15 and 16 are testing “System-based practice/Work in interdisciplinary teams”. Traditionally, in-person visits allow for patients to receive appointments for imaging studies and blood work prior to the completion of their visit. However, with telemedicine, the residents need to coordinate with nursing or clerical staff in order to schedule the imaging studies or blood work, and then one of the team members need to contact the patient again once those dates are confirmed. Questions 18 to 20 focus on “Practice-based Learning and Improvement” by asking trainees about their clinical experience and level of supervision trough telemedicine. Questions 23 to 25 assess the impact of telemedicine experience on internal medicine residents’ career plan.

### Statistical analysis

Statistical analysis was completed by using software MedCalc (version 14.12.0; Ostend, Belgium). The Chi-square test was used to examine (1) association between number of telemedicine visits during residency and career decision, and (2) association between number of telemedicine visits during residency and acceptance of the telemedicine practice as a permanent part of a future career. *P* <0.05 was considered indicative of a statistically significant difference.

## Results

Of 115 residents, 95 (82.6% of the residents) replied to this questionnaire. Before the COVID-19 pandemic, 5.3% (5/95) of residents had telemedicine experience outside the United States and 8.4% (8/95) had telemedicine experience within the United States (Table 1).

**Table 1:** Telemedicine experience of Internal Medicine Residents: Demographics, practice characteristics and career choice (n = 95).

	Percentage	Number
**Gender**		
Male	55.8%	53
Female	42.1%	40
Prefer not to answer	2.1%	2
**PGY level**		
PGY 1	38.9%	37
PGY 2	33.7%	32
PGY 3	27.4%	26
**Telemedicine experience before COVID pandemic**		
Outside United States	5.3%	5
Within United States	8.4%	8
**Number of attempted phone call per patient visit (within 3.5 hours)**		
1	13.7%	13
2 to 3 times	81%	77
4 or more	5.3%	5
**Number of telemedicine appointments (within 3.5 hours)**		
1–3 visit	45.3%	43
4–5 visit	42.1%	40
6–8 visit	12.6%	12
**Preference of practice in residency training**		
In-person visit	83.1%	79
Telemedicine	9.5%	9
No preference	7.4	7
**Does Telemedicine affect your career decision?**		
Yes	65.3%	62
No	8.4%	8
No difference	26.3%	25
**Acceptance of practice telemedicine when become attending physician**		
More than 50%	5.3%	5
Less than 50%	67.4%	64
No preference	6.3%	26

PGY: postgraduate year.

The survey shows that 81% (77/95) of residents will attempt to phone call 2 to 3 times if the patient did not pick up initially while 13.7% (13/95) will only attempt once. Currently, 42.1% (40/95) of trainees are able to conduct 4 to 5 telemedicine visits during 3.5 hours of clinic session while 45.3% (43/95) are able to perform 1 to 3 telemedicine visits. It is worth mentioning that, in our facility, each service distributes telemedicine visits differently. Some services give residents the whole clinic session to do telemedicine visits while some services mix telemedicine and in-person visits during the same clinic session. In the latter clinical scenario, residents need to find spare time between in-person visits to conduct the telemedicine visits.

Compared to in-person visits, a majority of residents responded that they received less clinical experience, less interaction with attendings and less supervision when engaging in telemedicine (Table 2). In addition, residents revealed feeling less confident managing chronic diseases through telemedicine visits in general. Only 2.1% (2/95) of trainees felt the patient received the same level of care when comparing telemedicine visits with in-person visits. None of the residents felt that the patients were always comfortable to discuss their medical condition through telephone and 73.6% of residents (70/95, citing their answer as always/often/sometimes) agreed that telemedicine visits increase the chance of patients lost to follow up. Medication reconciliation and language barrier were two main issues cited by residents.

**Table 2:** Telemedicine experience of Internal Medicine Residents: Resident satisfaction (n = 95).

**Practice based learning and improvement**	**Always**	**Often**	**Sometimes**	**Rarely**	**Never**
Do you feel that you earn the same clinical experience by telemedicine compared with in-person visit?	3.2% (3)	22.1% (21)	42.1% (40)	27.3% (26)	5.3% (5)
Do you feel that you have the same amount of attending supervision during telemedicine compared with in-person visits?	16.8% (16)	33.7% (32)	31.6% (30)	15.8% (15)	2.1% (2)
Do you feel that your supervising attending spends the same time discussing the case during telemedicine compared with in-person visits?	10.5% (10)	27.3% (26)	49.5% (47)	9.5% (9)	3.2% (3)
**Resident confidence**	**Always**	**Often**	**Sometimes**	**Rarely**	**Never**
Do you feel confident managing hypertension through telemedicine compared with in-person visit?	8.4% (8)	43.1% (41)	31.6% (30)	13.7% (13)	3.2% (3)
Do you feel confident managing heart failure/ CAD through telemedicine compared with in-person visit?	1.1% (1)	26.3% (25)	33.7% (32)	34.7% (33)	4.2% (4)
Do you feel confident managing DM through telemedicine compared with in-person visit?	5.3% (5)	26.3% (25)	34.7% (33)	30.5% (29)	3.2% (3)
Do you feel confident managing COPD / asthma through telemedicine compared with in-person visit?	4.2% (4)	34.7% (33)	29.5% (28)	28.4% (27)	3.2% (3)
Do you feel confident managing CKD through telemedicine compared with in-person visit?	2.1% (2)	33.7% (32)	36.8% (35)	24.2% (23)	3.2% (3)
**System-based practice/Work in interdisciplinary teams**	**Always**	**Often**	**Sometimes**	**Rarely**	**Never**
My telemedicine patient did not come to receive blood work or medication injection/infusion.	2.1% (2)	27.4% (26)	58.9% (56)	8.4% (8)	3.2% (3)
My telemedicine patient did not come to hospital to receive imaging study.	2.1% (2)	29.5% (28)	52.6% (50)	14.7% (14)	1.1% (1)
**Patient-Centered Care from Resident Standpoint**	**Always**	**Often**	**Sometimes**	**Rarely**	**Never**
Experience patient did not answer the phone.	4.2% (4)	52.6% (50)	35.8% (34)	6.3% (6)	1.1% (1)
Do you feel that patients are not comfortable in discussing their medical conditions to you via telephone?	0% (0)	27.4% (26)	37.9% (36)	22.1% (21)	12.6% (12)
Do you feel that telemedicine causes a larger language barrier between you and your patient (even with phone interpreter) compared to in person clinic visits?	16.8% (16)	36.9% (35)	32.6% (31)	6.3% (6)	7.4% (7)
Do you feel difficulty to do medication reconciliation through telemedicine compared with in-person visit?	7.4% (7)	24.2% (23)	42.1% (40)	18.9% (18)	7.4% (7)
Telemedicine increase the amounts of patients lost to follow up.	4.2% (4)	32.6% (31)	49.5% (47)	12.6% (12)	1.1% (1)
Do you think that patients receive the same level of care during telemedicine visits compared with in-person visits?	2.1% (2)	34.7% (33)	47.4% (45)	12.6% (12)	3.2% (3)

CAD: coronary artery disease. DM: diabetes mellitus. COPD: chronic obstructive pulmonary disease. CKD: chronic kidney disease.

This survey showed that 65.3% (62/95) of residents felt that the telemedicine experience will affect their career choice, while 26.3% (25/95) of residents did not feel affected by this experience. However, 83.1% (78/95) trainees preferred in-person clinic visits during their training and 67.4% (64/95) would prefer less than 50% of visits to be telemedicine in their future careers (Table 1).

We used Chi-Square test to examine (1) association between amount of telemedicine during residency and career decision as well as (2) association amount of telemedicine during residency and acceptance of the practice telemedicine when become attending physician. There were no statistically significant differences noted (p = 0.46 and p = 0.38, respectively). The amount of telemedicine conducted by residents seems to not affect career decisions of residents nor acceptance of the practice of telemedicine.

The result of this survey (Tables 1 and 2) and introduction of ACGME telemedicine regulation were presented to all faculty members and residents at a departmental conference. We emphasized that all faculty members should review the cases prior to appointments, in order to avoid complicated cases which are not suitable for telemedicine. We discussed “ACGME Common Program Requirements (Residency)” with all faculty and emphasized the supervision of all residents during telemedicine encounter [9].

Residents also received notification when the lab/imaging order was not completed by patients through the electronic medical system, EPIC, in a timely manner. Residents are able to send messages to the clinic nursing team to facilitate sending a reminder to patients.

## Discussion

The COVID-19 pandemic affected physician training tremendously, in all specialties, not only limited to Internal Medicine. Fortunately, we live in an age where technology is easily accessible, and sharing of information, such as conference and educational material, can be done in a timely manner. The effects of COVID-19 within the health care system have been separated into several fields such as patient care, medical education, and telemedicine [11–13]. Telemedicine provides continuity of care for patients while being able to avoid leaving their home, and has been used widely in the United States [14, 15]. ACGME promptly permitted resident involvement in telemedicine in an attempt to minimize education disruption. This will be permitted through the entire 2020–2021 academic year and further extension of this permission is expected.

On the other hand, ACGME acknowledged that “Traditional time-based or volume-based measures may not be fully achievable during this period. Educational experiences may be modified or disrupted through alternative forms of education, such as virtual learning, deployment to another clinical rotation or activity, or by missing a traditionally required rotation” [16]. The integration of telemedicine into residency training was not smooth, and several unanswered questions remain. First, if telemedicine will be a permanent component to residency training programs, the learning goals and expectations of telemedicine need to be established. For example, how much telemedicine clinic experience is appropriate for a residency program, specifically the number of patients a resident should see during one clinic session and if clinic sessions should be exclusively dedicated to telemedicine visits versus a hybrid clinic session with a mixture of in-person and telemedicine visits. Second, even though telemedicine supervision is defined by ACGME, every institution should publish its own guidelines and monitoring methods to ensure the resident receives adequate supervision and safety during training.

In this survey, 65.3% (62/95) of trainees reported telemedicine experience affected their career decision and 67.4% (64/95) of trainees would prefer less than 50% of telemedicine in their future practice. It seems like the telemedicine experience in our program made residents unwilling to practice telemedicine after graduation, but it is worth to know that 26.3% (25/95) reported that the telemedicine experience made no difference on their career decision. Potentially, high volume of telemedicine visits within 3.5 clinic hours could be the most important factor that made our residents dislike telemedicine. However, there is no statistically significant difference on the result. The appropriate patient volume of telemedicine during residency training remains unclear and further evaluation is needed to see how the telemedicine experience during residency affects resident career decision.

Interestingly, even though telemedicine was not common in residency training prior to this pandemic, the United Stated Medical License Examination (USMLE) step 2 Clinical Skills (CS) already introduced telemedicine to examinees [17]. Among 12 cases on the CS exam, examinees potentially will face 1 case of a telephone encounter, usually the case scenario will be the patient’s family phone call for their newborn child. We believe that our residents are not naïve to telemedicine because they practiced telemedicine when preparing for the CS exam. However, CS exam was currently suspended and replaced by 5 pathways due to COVID-19 pandemic.

There are several limitations to this survey. First, this is a single center survey solely within the department of Internal Medicine, and therefore may not be representative of residents in other specialties or other institutions. Second, telemedicine is a new concept to our facility and residents. More time may be needed to let trainees and trainers adapt to the new educational system. However, this survey does help to shed light on the areas that need to be further developed. Third, several questions in this survey did not fit only one domain of ACGME Core Competencies and not every domain of ACGME Core Competencies is covered by our 25-question survey. Standardized surveys will be needed for quality improvement measurement. Fourth, although this survey mainly focusses on trainees, lack of patient input limits the correlation that can be made between trainee perception of patient experience versus patients’ report of their experience. Fifth, telephone visits are only one type of telemedicine and residency training may benefit from other telecommunication technique.

Telemedicine has a large effect on residency training in terms of not only patient care, but resident education and career choice. Since telemedicine will likely become the norm in the foreseeable future, long term monitoring to ensure adequate resident training is warranted and follow up is needed to monitor how telemedicine affects residents’ learning experience or ultimate career plan.

## Supporting information

S1 Data Telemedicine experience of Internal Medicine resident in NYC in COVID-19 era. (DOCX)
