# Supplementary material for: Telemedicine experience of NYC Internal Medicine residents during COVID-19 pandemic
Source: PLoS One. 2021 Feb 8;16(2):e0246762. doi: 10.1371/journal.pone.0246762 (PMC7869991; doi:10.1371/journal.pone.0246762)
Supplement: S1 Data — (DOCX) [file pone.0246762.s001.docx]

**Supplement 1**

Telemedicine Experience of Internal Medicine Resident in NYC in COVID-19 era

1. Do you identify as

[ ] Male
[ ] Female

[ ] Prefer not to answer

2) What PGY level are you?
[ ] PGY 1
[ ] PGY 2
[ ] PGY 3

3) Have you ever participated in telemedicine visits for clinic patients before COVID-19 crisis in a country **OUTSIDE the USA**?
[ ] Yes
[ ] No

4) Have you ever participated in telemedicine visits for clinic patients before COVID-19 crisis **WITHIN the USA**?
[ ] Yes
[ ] No

5) Do you feel that patients are not comfortable in discussing their medical conditions to you via telephone?
[ ] Always
[ ] Often

[ ] Sometimes

[ ] Rarely

[ ] Never

6) Do you feel that telemedicine causes a larger language barrier between you and your patient (even with phone interpreter) compared to in person clinic visits?
[ ] Always

[ ] Often

[ ] Sometimes

[ ] Rarely

[ ] Never

7) Have you ever experienced your patient not picking up the telephone?

[ ] Always

[ ] Often

[ ] Sometimes

[ ] Rarely

[ ] Never

8) During your 3.5 hours clinic session, how many times you will attempt to call a patient?

[ ] I will call only one time
[ ] 2 times
[ ] 3 times
[ ] 4 or more

9) Do you feel difficulty to do **medication reconciliation** through telemedicine compared with in-person visit?

[ ] Always

[ ] Often

[ ] Sometimes

[ ] Rarely

[ ] Never

10) Do you feel confident managing **hypertension** through telemedicine compared with in-person visit?
[ ] Always

[ ] Often

[ ] Sometimes

[ ] Rarely

[ ] Never

11) Do you feel confident managing **heart failure/CAD (coronary artery disease)** through telemedicine compared with in-person visit?
[ ] Always

[ ] Often

[ ] Sometimes

[ ] Rarely

[ ] Never

12) Do you feel confident managing **diabetes** through telemedicine compared with in-person visit?
[ ] Always

[ ] Often

[ ] Sometimes

[ ] Rarely

[ ] Never

13) Do you feel confident managing **COPD (chronic obstructive lung disease) /asthma** through telemedicine compared with in-person visit?
[ ] Always

[ ] Often

[ ] Sometimes

[ ] Rarely

[ ] Never

14) Do you feel confident managing **CKD (chronic kidney disease)** through telemedicine compared with in-person visit?

[ ] Always

[ ] Often

[ ] Sometimes

[ ] Rarely

[ ] Never

15) My telemedicine patient did not come to **receive blood work or medication injection/infusion**.
[ ] Always

[ ] Often

[ ] Sometimes

[ ] Rarely

[ ] Never

16) My telemedicine patient did not come to hospital to **receive imaging study** (mammogram, X-ray, CT scan, MRI scan, etc.)

[ ] Always

[ ] Often

[ ] Sometimes

[ ] Rarely

[ ] Never

17) Telemedicine increase the amounts of patients lost to follow up.
[ ] Always

[ ] Often

[ ] Sometimes

[ ] Rarely

[ ] Never

18) Do you feel that you have the same **amount of attending supervision** during telemedicine compared with in-person visits?
[ ] Always receive supervision (100%)

[ ] Often receive supervision (75%)

[ ] Sometimes receive supervision (50%)

[ ] Rarely receive supervision (25%)

[ ] Never receive supervision (0%)

19) Do you feel that your supervising attending spends the same time **discussing the case during telemedicine** compared with in-person visits?

[ ] Always discuss the case (100%)

[ ] Often discuss the case (75%)

[ ] Sometimes discuss the case (50%)

[ ] Rarely discuss the case (25%)

[ ] Never discuss the case (0%)

20) Do you feel that you earn the same **clinical experience** by telemedicine compared with in-person visit?

[ ] The same level of clinical experience (100%)

[ ] Often earn clinical experience (75%)

[ ] Sometimes earn clinical experience (50%)

[ ] Rarely earn clinical experience (25%)

[ ] Did not earn any clinical experience (0%)

21) Do you think that patients receive the same **level of care** during telemedicine compared with in-person visits?
[ ] the same level (100%)

[ ] 75%

[ ] 50%

[ ] 25%

[ ] 0%

22) How many telemedicine visits are you able to do during your 3.5 hours clinic time?
[ ] 1-3

[ ] 4-5

[ ] 6-8

23) Compared with telemedicine visits, I prefer doing in-person visits.
[ ] True
[ ] False

[ ] There is no difference for me

24) Do you think telemedicine will affect your future career/job decisions?
[ ] Yes
[ ] No

[ ] There is no difference for me

25) When you looking for a primary care physician job / outpatient physician job, what percent of telemedicine is appropriate in your practice?

[ ] 100%

[ ] 75%

[ ] 50%

[ ] 25%

[ ] 0%

[ ] There is no difference for me
